# Variability in Glycemic Control with Temperature Transitions during Therapeutic Hypothermia

**DOI:** 10.1155/2017/4831480

**Published:** 2017-09-18

**Authors:** Krystal K. Haase, Jennifer L. Grelle, Faisal A. Khasawneh, Chiamaka Ike

**Affiliations:** ^1^Division of Adult Medicine, Department of Pharmacy Practice, Texas Tech University Health Sciences Center School of Pharmacy, 1300 S. Coulter St., Room 206, Amarillo, TX 79106, USA; ^2^Advanced ICU Care, Houston Office, 7505 S. Main St., Houston, TX 77030, USA

## Abstract

**Purpose:**

Patients treated with therapeutic hypothermia (TH) and continuous insulin may be at increased risk of hyperglycemia or hypoglycemia, particularly during temperature transitions. This study aimed to evaluate frequency of glucose excursions during each phase of TH and to characterize glycemic control patterns in relation to survival.

**Methods:**

Patients admitted to a tertiary care hospital for circulatory arrest and treated with both therapeutic hypothermia and protocol-based continuous insulin between January 2010 and June 2013 were included. Glucose measures, insulin, and temperatures were collected through 24 hours after rewarming.

**Results:**

24 of 26 patients experienced glycemic excursions. Hyperglycemic excursions were more frequent during initiation versus remaining phases (36.3%, 4.3%, 2.5%, and 4.0%, *p* = 0.002). Hypoglycemia occurred most often during rewarming (0%, 7.7%, 23.1%, and 3.8%, *p* = 0.02). Patients who experienced hypoglycemia had higher insulin doses prior to rewarming (16.2 versus 2.1 units/hr, *p* = 0.03). Glucose variation was highest during hypothermia and trended higher in nonsurvivors compared to survivors (13.38 versus 9.16, *p* = 0.09). Frequency of excursions was also higher in nonsurvivors (32.3% versus 19.8%, *p* = 0.045).

**Conclusions:**

Glycemic excursions are common and occur more often in nonsurvivors. Excursions differ by phase but risk of hypoglycemia is increased during rewarming.

## 1. Introduction

Cardiac arrest is associated with high mortality rates and has few proven treatment modalities [1]. Therapeutic hypothermia (TH) is associated with improved neurologic outcomes and decreased mortality and is now recognized as standard of care for cardiac arrest patients [2–5].

While therapeutic hypothermia has multiple benefits, the temperature fluctuations encountered during this process cause clinically significant physiologic changes. The effect of temperature variation on glycemic control has been identified as a potential complication. Hypothermia reduces insulin secretion and increases insulin resistance in animal models and in cardiopulmonary bypass patients [6–12]. The temperatures used in these studies (<28°C) were much lower than those currently used in therapeutic hypothermia.

The impact of TH on the magnitude and significance of variation in glycemic control is unclear. Clinically significant differences in glucose, glucose variability, and insulin utilization have been reported in TH patients during hypothermia versus after rewarming [13]. Alternatively, these differences have been correlated to the severity of stress soon after arrest rather than to hypothermic temperatures [14]. Evaluation of changes in glycemic control during temperature transitions between hypothermia and normothermia may be particularly relevant to this question. Both sustained hyperglycemia and hypoglycemia are associated with increased mortality in TH patients [15]. Glucose variability is also a predictor of increased mortality in TH and other patient groups [13, 16–18]. To the best of our knowledge, rates of glycemic excursions and glucose variability have not been characterized during the transitional temperature phases.

The aim of this pilot study was to assess the frequency and severity of glycemic excursions, glucose variability, and insulin requirements during the transitional temperature phases of TH. We also sought to determine the relationship between glycemic control patterns and survival.

## 2. Materials and Methods

### 2.1. Patients

We identified all patients admitted to the intensive care units (ICUs) of Northwest Texas Hospital, Amarillo, Texas, between January 2010 and June 2013, who had sudden cardiac arrest treated with TH. Inclusion criteria were (1) diagnosis of cardiac or cardiorespiratory arrest (ICD-9  427.5) with return of spontaneous circulation treated with TH, (2) concurrent treatment with protocol-based continuous infusion insulin therapy, and (3) age ≥ 18 years. We limited inclusion to patients admitted after the date of implementation of a comprehensive TH protocol at our institution. Patients were excluded if they did not complete the 24-hour TH protocol for any reason. Prisoners or wards of the state were also excluded. All patients were cooled to a target temperature of 33°C for 24 hours after return of spontaneous circulation (ROSC) using internal and external cooling methods and then rewarmed passively in a manner consistent with guidelines that were current at the time of study [1]. The study received approval from both the hospital and university Institutional Review Boards.

### 2.2. Blood Glucose Management

Glycemic management with continuous infusion insulin was standardized for most patients based on the modified Atlanta protocol [19]. Physicians could opt for a simplified continuous infusion regimen with linear dosage changes at any time during therapy. Arterial blood glucose (BG) was measured hourly using a Venous Arterial blood Management Protection (VAMP) system and immediately analyzed using point-of-care testing. This closed blood sampling system allows for frequent measurement of accurate samples with reduced blood waste and infection risk. Abnormal readings were confirmed through central laboratory analysis. Patients receiving the simplified regimen could transition to 2-hour glucose measurements once stable. The target BG range of 7.77–10 mmol/L was achieved using calculated titration based on the degree of response to the previous insulin rate. The use of exogenous dextrose-containing solutions was minimized. Nutrition was typically withheld until the completion of the TH protocol. Continuous insulin infusion was discontinued when it was no longer required to maintain target glucose concentrations.

### 2.3. Data Extraction

Demographic data, including baseline characteristics, comorbidities, cardiac arrest-related factors, treatment interventions, and complications of glycemic excursions, were collected manually from patient medical records. Blood glucose measures and insulin infusion rates were collected from standard continuous infusion protocol documentation logs. Hourly temperatures were likewise collected from therapeutic hypothermia protocol documentation records and matched with corresponding glucose and insulin data.

Data were collected for four separate time periods for each patient: Phase 1: initiation (time from initiation of cooling to achievement of target temperature), Phase 2: hypothermia (24 hours following achievement of 33°C), Phase 3: rewarming (time from discontinuation of TH to attainment of target temperature of 36.5°C), and Phase 4: normothermia (24 hours following rewarming). Frequency of glycemic excursions was calculated for each phase as a percentage of total glucose measures for the time period. Prevalence of glycemic excursions per phase was also determined as this value is less affected by frequency of glucose measures. Hyperglycemic excursions were defined as BG > 10 mmol/L and hypoglycemic excursions were defined as BG < 3.9 mmol/L. Hypoglycemia was considered severe if the patient exhibited symptoms, including seizure, or had a BG < 2.22 mmol/L. Insulin infusion requirements per phase were reported as mean units/hr. Glucose variation was reported as delta BG, the difference between the highest and lowest BG readings during each phase. Coefficient of glucose variation (SD/mean × 100%) was also calculated, as this parameter normalizes variability for different mean glucose values and has been independently associated with mortality in critically ill patients [18].

The primary outcome was frequency of glycemic excursions in each phase of TH. Secondary outcomes included changes in mean glucose, glucose variation, coefficient of glucose variation, severity of glycemic excursions, and insulin requirements during each phase of TH. We also determined whether these indices correlated with in-hospital mortality.

### 2.4. Statistical Analysis

Glycemic excursions were expressed as a percentage of total measures for each phase. Continuous variables not normally distributed are reported as medians and ranges. The Skillings-Mack test was used for comparisons across the four phases to address missing glucose and insulin data for some subjects in Phase 1 who were cooled prior to arrival and in Phase 4 who were no longer on insulin protocol [19]. Univariate associations with survival outcomes were determined using Chi-square, Fisher's Exact, Mann–Whitney *U*, or Student's *t*-test as appropriate. Two-tailed tests of significance were used, and *p* < 0.05 was considered significant. Statistics were evaluated using XLSTAT Software version 2013.2.01 (Addinsoft, New York, NY).

## 3. Results

### 3.1. Patient Characteristics

Thirty-two of the 48 patients (66.7%) who were treated with TH after cardiac arrest received continuous infusion insulin therapy. Twenty-six patients met inclusion and exclusion criteria. Four patients were excluded from the primary analysis because they did not complete the 24-hour TH protocol. Two additional patients were excluded because of transfer from prison facility and inconclusive evidence of cardiac arrest, respectively. The mortality rate in this cohort was similar to the overall documented mortality rate for patients receiving TH during the same time period (50% versus 51.1%, *p* = 1). The 26 patients included in the analysis had a total of 904 distinct glucose/insulin/temperature measurements. Patient characteristics are summarized in Table 1. The majority of arrests were of cardiac origin (80%) with acute coronary syndrome described as the most common etiology. No arrests were attributed to glucose-related conditions (i.e., diabetic ketoacidosis, hypoglycemia).

### 3.2. Glycemic Control

**Figure 1:**
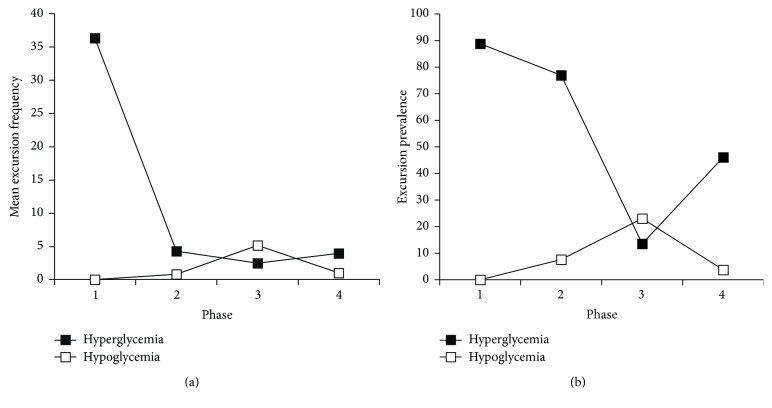
Glycemic excursions during each phase of therapeutic hypothermia. (Phase 1: initiation, Phase 2: hypothermia, Phase 3: rewarming, Phase 4: normothermia).

Glycemic excursions, as illustrated in Figure 1, occurred in the majority of patients (24 of 26, 92.3%). Frequency of hyperglycemia as a percent of total measures was highest during initiation when compared to other phases (*p* = 0.002). Hyperglycemia was most prevalent during initiation and hypothermia with significant declines during rewarming and normothermia (*p* = 0.01). Hypoglycemia was infrequent in relation to total glucose measures. However, more patients experienced hypoglycemic excursions during rewarming when compared to other phases (*p* = 0.02). Neither severe hypoglycemia (BG < 2.22 mmol/L) nor seizures were documented in any phase. Overall and phase-specific frequency of excursions did not differ significantly based on presence or absence of diabetes.

**Figure 2:**
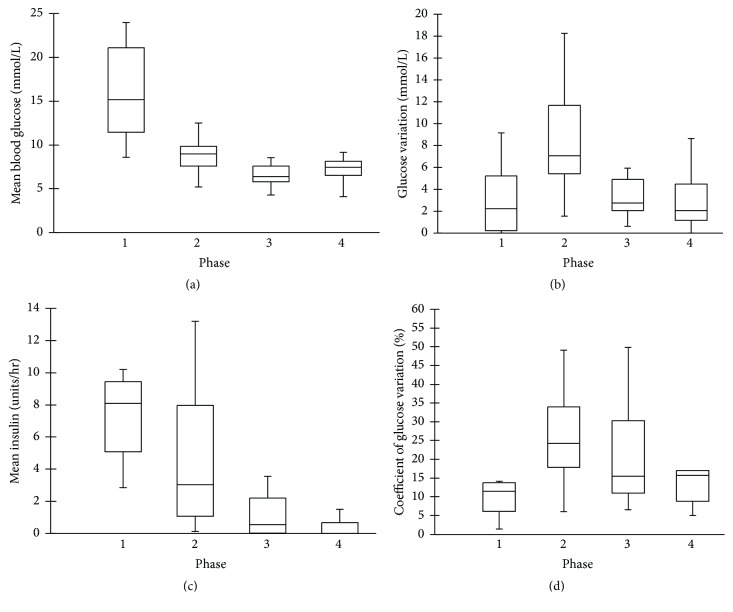
Average glycemic parameters per phase. The* lines within the boxes* represent medians, the* boxes* represent the 25th and 75th percentiles, and the* whisker lines* represent the 5th and 95th percentiles. (a) Mean blood glucose (BG) per phase (mmol/L). *p* < 0.001. (b) Glucose variability (mmol/L) = BGmax − BGmin per phase, *p* = 0.001. (c) Mean insulin dose per phase (units/hr), *p* < 0.001. Data only available for nine patients for Phase 1 due to out-of-hospital cooling. (d) Percentage coefficient of glucose variation (%) = BG standard deviation/BG mean *∗* 100 per phase, *p* = 0.097.

Mean BG, glucose variation, and coefficient of glucose variation per phase are represented in Figure 2. Mean BG varied significantly across phases but was highest during initiation (*p* < 0.001). In contrast, mean glucose variation was significantly higher during hypothermia when compared to other phases (*p* = 0.001). Coefficient of glucose variation also trended higher during hypothermia but the difference was not statistically significant (*p* = 0.1). Average glucose values did not differ between patients with versus without diabetes (*p* = 0.96).

### 3.3. Insulin Requirements

Seventeen patients were managed solely using the modified Atlanta protocol. The remaining nine patients were transitioned to a simplified protocol consisting of BG checks a minimum of every two hours with linear changes in insulin dose. The median per-patient average insulin requirements (Figure 2(c)) were higher during induction and hypothermia (8.2, 3.0, 0.5, and 0 units/hr, *p* < 0.001). Insulin requirements decreased, on average, by 37% per patient between hypothermia and rewarming and an additional 13% during normothermia. Patients with hypoglycemic excursions received higher insulin doses prior to rewarming than those without hypoglycemia (16.2 versus 2.1 units/hr, *p* = 0.03). History of diabetes was not associated with significantly higher insulin requirements. Specifically, the median average insulin rate during hypothermia phase was 3.82 units/hr in patients with diabetes versus 1.89 units/hr in those without diabetes (*p* = 0.13).

### 3.4. Patient Outcomes

Thirteen patients survived to hospital discharge (50%). Age, sex, admission location, ROSC, and type of arrest were no different between survivors and nonsurvivors (Table 1).  Table 2 depicts differences in glucose trends between survivors and nonsurvivors. Frequency of excursion as a percent of total glucose measures was significantly higher in nonsurvivors than survivors (*p* = 0.045). First measured glucose and glucose variation by delta BG also trended higher in nonsurvivors. Average glucose and coefficient of glucose variation were similar between groups. Fewer patients in the survivor group required dextrose administration based on hypoglycemia protocol. However, these results were not statistically significant (*p* = 0.1).

## 4. Discussion

We sought to quantify the changes that occur in glycemic control during temperature transitions in post-cardiac arrest patients by evaluating the frequency of glycemic excursions and glucose variation within all phases of TH. We found that the types and frequency of glycemic excursions change through each phase of TH and that meaningful differences do occur during the transitional phases. Hyperglycemia was predominant during both induction and hypothermia and hypoglycemia was most prevalent during rewarming. Insulin requirements also varied through all four phases with patients requiring higher doses during induction. Insulin dose prior to rewarming was an important risk factor for hypoglycemic events. This work suggests that glycemic excursion frequency and glucose variation may differ in survivors versus nonsurvivors in this patient population.

A previous study by Cueni-Villoz and colleagues described significant differences in mean BG, BG variability, and mean insulin dose during the maintenance phase of TH when compared to normothermia [13]. This study suggests, but does not describe, the changes that occur between each of the phases, particularly during rewarming. Dramatic reductions in insulin requirements during the rewarming phase could lead to hypoglycemia and associated negative consequences such as poor neurologic outcome [20]. In our analysis, hypoglycemia was three times more likely to occur during rewarming than during hypothermia. Patients receiving high doses of insulin prior to rewarming were at the greatest risk of hypoglycemia during the rewarming phase. Rate of rewarming varied considerably and was not associated with hypoglycemia risk. Use of the modified Atlanta protocol for continuous insulin therapy minimized the overall risk of severe or prolonged hypoglycemia. However, our findings suggest that more significant reductions should be made to insulin doses prior to beginning the rewarming process.

Animal models suggest that glycemic excursions are most likely to occur in patients cooled to lower temperatures and thus more conservative temperature targets may reduce the risk of these events [6–10]. Nielsen et al. found that patients cooled to 36°C following cardiac arrest had similar survival and neurological outcomes compared to those cooled to a target of 33°C [21]. Patients cooled to 33°C had a higher prevalence of serious adverse events. However, no difference was noted in the occurrence of hypoglycemia. Other glycemic indicators were not reported. In our study, there was no specific temperature threshold or change in temperature that was associated with hypoglycemic risk. Further investigation is warranted to determine whether target temperature has a significant impact on glycemic indicators.

Previous studies are conflicting regarding the relationship between hyperglycemia and TH. In the previously mentioned study, the authors identified an association between TH and higher BG levels and glucose variability [13]. However, a study by Ettleson and colleagues showed no independent effect of TH on BG levels and attributed hyperglycemia to the severity of stress from cardiac arrest [14]. In our analysis, significant hyperglycemia did occur prior to cooling, yet temperature-dependent changes were also noted, particularly with rewarming. This suggests that hyperglycemia following cardiac arrest in patients treated with TH is due to both stress- and temperature-related factors.

Glucose variability has been shown to correlate with mortality in critically ill patients independent of mean glucose and frequency of hypoglycemic events [17, 18]. Increased glucose variability has been theorized to be secondary to changes in homeostasis due to critical illness [18]. However, specific mechanisms have not been elucidated. A variety of methods have been utilized to calculate glucose variability. We chose to measure delta BG because of the previous use of this parameter in assessing glycemic control during TH and because it is less significantly impacted by variation in number of BG measures. We also calculated standard deviation given its common usage and percent coefficient of glucose variation as this parameter is normalized to account for different mean glucose values. In our analysis, glucose excursion frequency was more predictive of survival status than any of the variation parameters, though delta BG trended toward significance. However, given the exploratory nature of this study, the results should be interpreted as hypothesis-generating and warrant validation in a larger patient population.

We acknowledge several limitations to this study. The study included a limited number of subjects. The sample was adequate for our primary aim, comparisons of between-phase differences in excursions, glycemic control, and insulin rates. However, the study also identified several potentially meaningful differences in relation to glycemic control and patient survival. These results were only exploratory in nature as we were unable to assess the impact of covariates on secondary outcomes. This was a retrospective cohort study conducted at a single center. The addition of other sites could have increased sample size and robustness. However, the authors were unaware of any other site that utilized similar glucose management procedures and documentation systems. Limiting to a single institution provided homogeneity in terms of protocol-based glucose management as well as a unified approach to TH in our patients. These data represent the outcomes from a typical, medium-sized, tertiary care facility but may not be generalizable to other institutions. This study would be difficult to conduct prospectively because of issues with rapid subject identification and consent. Because of the retrospective nature of the study, some data points, such as baseline characteristics, were missing for some patients. In addition, individual BG measurements were not always documented for phases 1 and 4. This was attributed to several factors. A number of patients were cooled prior to hospital admission or initiation of insulin infusion. In addition, glucose management was deescalated for several patients who improved significantly prior to return to normothermia. Once a patient had been taken off of or no longer required continuous insulin therapy, glucose measures were not recorded. Our analysis used the Skillings-Mack test, a paired, nonparametric ANOVA that is capable of handling missing data points in the sample [22]. Finally, this study was only able to evaluate differences in documented in-hospital patient outcomes. We were not able to assess other major outcomes from cardiac arrest that have been associated with glycemic control, including long-term neurologic recovery.

## 5. Conclusions

Glycemic excursions are common during TH and are phase-specific. Higher glucose values and insulin requirements during both induction and hypothermia suggest the presence of a combination of stress- and temperature-associated changes that warrant further study. Hypoglycemia can occur during rewarming and is most problematic in patients who require higher insulin doses during hypothermia. Careful monitoring and titration of insulin therapy is warranted in TH patients, particularly during rewarming phase.

## Figures and Tables

**Table 1:** Baseline characteristics.

	Overall *n* = 26	Survivors *n* = 13	Nonsurvivors *n* = 13	*p*
Age, yrs	66 (35–86)	65 (35–84)	73 (42–86)	0.40
Male (%)	17 (65.4)	4 (69.2)	5 (61.5)	1.00
Race (%)				
White	19 (73.1)	11 (84.6)	8 (61.5)	0.39
Black	4 (15.4)	1 (7.7)	3 (23.1)	
Other/unknown	3 (11.5)	1 (7.7)	2 (15.4)	
Transfer from outside facility (%)	5 (19.2)	2 (15.4)	3 (23.1)	1.00
Minutes to return to spontaneous circulation^a^	11 (4–83)	10 (4–33)	21 (2–83)	0.15
Arrest etiology, cardiac (%)^b^	20 (80)	11 (84.6)	9 (75)	0.65
Initial arrest rhythm, ventricular fibrillation (%)^c^	14 (58.3)	9 (69.2)	5 (45.4)	0.41
History of diabetes (%)	14 (53.8)	5 (38.5)	9 (69.2)	0.24
Treatments (%)				
Vasopressor	24 (92.3)	12 (92.3)	12 (92.3)	1.00
Dextrose-containing fluids	6 (23.1)	3 (23.1)	3 (23.1)	1.00
Glucocorticoids	5 (19.2)	2 (15.4)	3 (23.1)	1.00
Enteral nutrition	1 (3.8)	0	1 (7.7)	1.00

^a^Data available for 80.8% of cohort; ^b^arrest cause not documented in 1 nonsurvivor; ^c^arrest rhythm not documented for 2 nonsurvivors. Nonparametric data as median (range).

**Table 2:** Differences between survivors and nonsurvivors.

	Survivors *n* = 13	Nonsurvivors *n* = 13	*p*
Average measured glucose, mmol/L	9.16 ± 4.08	9.38 ± 2.24	0.844
First measured glucose, mmol/L	12.21 ± 6.26	15.66 ± 4.65	0.062
Glucose variation (max-min), mmol/L	9.16 ± 6.15	13.38 ± 6.13	0.092
Percentage coefficient of glucose variation	31.9 ± 18	35.4 ± 11	0.562
Frequency of excursion	3 (0–27)	9 (2–28)	0.057
Excursions as percent of total measures	7 (7–77)	25 (8–70)	0.045
Required hypoglycemia protocol (%)	2 (15.4)	7 (53.8)	0.097

Continuous variables are expressed as mean ± SD and nonparametric data as median (range).
